# Clinical performance of indirect hybrid ceramic onlay restorations cemented with injectable resin composite versus dual-cure resin cement: an 18-month randomized clinical trial

**DOI:** 10.1186/s12903-025-06903-5

**Published:** 2025-09-23

**Authors:** Hamsa Ashraf, Adel El Tannir, Ahmed El Zohairy, Dina Kamal

**Affiliations:** 1 Conservative Dentistry Department, Faculty of Dentistry, Cairo University, Cairo, Egypt; 2 Fixed Prosthodontics Department, Faculty of Dentistry, Cairo University, Cairo, Egypt

**Keywords:** Injectable resin composite, Dual-cure resin cement, Hybrid ceramics, Onlay, Indirect restoration, Cementation, USPHS criteria

## Abstract

**Background:**

The cementation of indirect restorations is a critical step influencing their long-term clinical success. While dual-cure resin cements are widely used, injectable resin composites have emerged as promising alternatives, offering improved handling, high filler content, and esthetic stability. However, their clinical performance as luting agents for indirect restorations remains insufficiently explored. This trial assessed the clinical performance of indirect hybrid ceramic onlay restorations cemented with injectable resin composite versus dual-cure resin cement over 18 months.

**Materials and methods:**

A total of 28 participants were randomly assigned to two groups (*n* = 14) based on the cementation protocol. The intervention group received onlays cemented with injectable resin composite (BEAUTIFIL Flow Plus X F03, Shofu Dental Corporation), while the control group received dual-cure resin cement (BeautiCem SA, Shofu Dental Corporation). Standardized procedures were followed for cavity preparation, immediate dentin sealing, cavity optimization, impression-taking, onlay fabrication (SHOFU Block HC, Shofu Dental Corporation), and cementation. Restorations were evaluated at baseline, 6, 12, and 18 months using the modified USPHS criteria. Data were statistically analyzed with significance set at *P* ≤ 0.05. Intergroup comparisons were assessed with Chi-squared test, while intragroup comparisons were assessed with Cochran’s Q test. Kaplan–Meier analysis and the log-rank test were used to evaluate survival rates.

**Results:**

Inter- and intragroup comparisons revealed no significant differences between the two groups for most outcomes (*P* > 0.05). However, at 18 months, dual-cure resin cement exhibited significantly higher marginal discoloration than injectable resin composite (*P* = 0.038). Within the dual-cure resin cement group, a significant decline in alpha scores for marginal discoloration was observed from baseline to 18 months (*P* = 0.007). While no restorations failed (Charlie score), Kaplan–Meier survival analysis and the log-rank test demonstrated a statistically significant difference between the two groups (*P* = 0.029).

**Conclusions:**

After 18 months, injectable resin composite demonstrated acceptable and comparable clinical performance to dual-cure resin cement, with the added benefit of reduced marginal discoloration.

**Clinical relevance:**

Injectable resin composites offer a viable alternative to dual-cure resin cements for luting indirect restorations with enhanced esthetic outcomes.

**Trial Registration:**

https://clinicaltrials.gov/, (NCT05954156), 20-07-2023.

## Introduction

Among the various factors influencing the clinical success and longevity of indirect restorations, the cementation protocol serves as a key determinant. Achieving a durable bond is essential for preserving the structural integrity and long-term functionality of restorations [1]. Marginal integrity is particularly critical, as discrepancies may cause discoloration, microleakage, cement dissolution, and recurrent caries. The selection of a luting agent holds a pivotal role in minimizing these discrepancies, thereby impacting overall clinical outcomes [2].

Resin cements are widely used for their strong adhesion and compatibility with various restorative materials, including ceramics and zirconia. Based on their bonding mechanisms, resin cements are categorized as adhesive or self-adhesive, and their polymerization modes—chemical, light, or dual-cure—offer versatility in clinical applications [3]. While dual-cure cements are effective for thick or opaque restorations, light-cured cements are often preferred for their extended working time, which enhances procedural precision [4]. However, resin cements may have limitations related to filler content, mechanical properties, and chemical incompatibility with adhesives, potentially affecting their long-term durability [5, 6].

To address these limitations, light-polymerized restorative resin composites—originally developed for direct restorations—have been explored as alternative luting agents [7, 8]. These materials offer superior stain resistance, color stability, and mechanical properties. However, their higher filler content increases viscosity, potentially resulting in a thicker and less optimal cementation line at the adhesive interface [9]. Preheating techniques have been proposed to reduce viscosity and enhance adaptation, though there is no consensus on optimal devices, temperatures, or application protocols [8, 9]. Successful preheating depends on using calibrated, moisture-free devices for consistent temperature control, immediate application to minimize temperature loss, and selecting materials specifically formulated for preheating [9, 10].

Flowable resin composites have gained attention as luting agents due to their low viscosity, which facilitates optimal restoration seating [11, 12]. The advent of injectable resin composites has further simplified cementation by combining ease of application with enhanced properties, making them viable alternatives to preheated composites. Highly filled injectable composites share similarities with resin cements but offer superior mechanical performance and handling, positioning them as a promising option for cementing indirect restorations [13].

Despite these advancements, the clinical use of injectable composites for cementation remains underexplored. While several studies have investigated flowable composites as luting agents, no clinical trial to date has evaluated the performance of injectable resin composites for the cementation of indirect restorations. The trial was designed to address this gap by assessing the clinical viability of injectable composites in esthetically demanding, minimally invasive restorative scenarios. Specifically, this trial aimed to compare the clinical performance of hybrid ceramic onlays cemented with injectable resin composite versus dual-cure resin cement. The null hypothesis tested was that no significant difference would be observed between the two cementation techniques after an 18-month follow-up.

## Materials and methods

### Study design and setting

This is an 18-month, two-arm parallel, triple-blind randomized clinical trial with a 1:1 allocation ratio, conducted within a superiority framework. The trial was conducted at the clinics of the Conservative Dentistry Department, Faculty of Dentistry, Cairo University, From August 2023 to February 2025. A total of 28 eligible participants were randomly allotted to two groups (*n* = 14) according to the cementation protocol. To maintain the independence of observations and avoid intra-individual clustering, only one restoration per participant was included. Each participant contributed a single tooth to the trial, ensuring the tooth was both the clinical and statistical unit of analysis. One group received indirect onlay restorations cemented with injectable resin composite, while the other received restorations cemented with dual-cured resin cement. Restorations were assessed using the modified USPHS criteria at baseline and after 6, 12, and 18 months. All participants were thoroughly informed about the study and provided written informed consent. A comprehensive medical and dental history was obtained, followed by clinical examination, with findings documented in diagnostic charts. Prophylactic scaling and polishing were performed, and any pre-existing dental conditions were addressed prior to the commencement of the trial. Participants received oral hygiene instructions on proper brushing and flossing techniques, which were reinforced at each follow-up visit.

### Ethical approval and trial registration

All procedures compiled with the ethical standards of the 1975 Declaration of Helsinki and were approved by the Research Ethics Committee, Faculty of Dentistry, Cairo University (ID: 12723). The study adheres to the 2010 CONSORT reporting guidelines and is registered in the clinical trials registry database (https://clinicaltrials.gov/, ID: NCT05954156, 20-07-2023).

### Sample size calculation

Based on a previous study [14], the probability of achieving score A for restoration integrity using dual-cure resin cement was 0.99, and the probability of score B was 0.01. For the injectable resin composite (intervention), the estimated probabilities were 0.9 for score A and 0.1 for score B, aiming to detect a 10% difference. Using an alpha (α) level of 0.05 (5%) and a power of 80%, the required sample size (n) was calculated to be 22. To account for potential dropouts during follow-up, the sample size was increased by 30%, resulting in a total of 28 cases (14 per group). The calculation was performed using G*Power 3.1.9.2, employing the chi-square test.

### Eligibility criteria

The inclusion criteria comprised male or female participants aged 22–40 years with a vital, asymptomatic, broken-down lower molar exhibiting at least one missing wall and one or more weakened or absent cusps, classified as ICDAS score (5). All participants had natural opposing dentition; favourable occlusion; no history of high-stress dietary habits; no clinical signs of pulpal involvement; healthy periodontal status; and were in overall good general health. The exclusion criteria included individuals with systemic diseases or major health conditions; parafunctional habits; temporomandibular joint disorders; signs of pulp pathology or periapical pathosis; endodontically treated teeth; advanced or active periodontal disease; xerostomia, smoking; drug addiction; or any condition that could compromise study compliance.

### Randomization, sequence generation, and allocation concealment

A total of 28 eligible participants (mean age = 29.53 ± 4.60 years; 16 females, 12 males) were randomly allotted into two groups. Simple randomization was carried out using a random sequence generator (https://www.random.org/), generating numbers from 1 to 28. Participants assigned numbers 1–14 were allocated to the intervention group, while numbers 15–28 were allocated to the control group. To ensure allocation concealment, a contributor not involved in any other phase of the trial placed the numbers into opaque, sealed envelopes to keep the sequence hidden. This trial followed a triple-blind design, in which participants, assessors and statisticians were blinded to the materials used. However, due to differences in the materials’ presentation and application techniques, blinding the operator was not feasible.

### Clinical procedures

The materials used are summarized in Table 1. All materials were applied according to the manufacturers’ instructions, and all clinical procedures were performed by a single operator.

**Table 1:** Materials’ trade names, description, composition, manufacturers and batch numbers

Trade name	Description	Composition	Manufacturer	Batch number
Ventura Etching Gel	Etchant	Phosphoric acid (37%), Thickening agent (8%), Water (54.7%), Dispersing coloring agent (0.3%)	Madespa, S.A	1697–26485
BeautiBond Xtreme	Universal adhesive	Bis-GMA (10–20%), TEGDMA (< 10%), Acid monomer (< 20%), Acetone and water (65–85%), Silane coupling agent (< 5%), Others (< 5%)	Shofu Dental Corporation	122234
BEAUTIFIL Flow Plus X F03	Bioactive injectable nano-hybrid composite with “Giomer” filler technology	Bis-GMA (10–20%), TEGDMA (5–20%), Bis-MPEPP (5–15%), Aluminofluoro-borosilicate glass (50–60%), Silane coupling agent (5–10%), SiO_2_ (0.5-5%), Al_2_O_3_ (1–5%), Polymerization initiator, Pigments, Others	Shofu Dental Corporation	012418
BeautiCem SA	Self-etch, self-adhesive, dual-cure resin cement with “Giomer” filler technology	Paste A: UDMA (20–30%), Aluminofluoro-borosilicate glass (30–60%), Glass powder (1–10%), NPGDMA (1–10%), Silica (1–5%), Polymerization initiator, Others Paste B: UDMA (15–25%), 2-HEMA (10–20%), Carboxylic acid monomer (1–5%), Phosphonate monomer (1–5%), Zirconium siliate filler (40–60%), Silica (1–5%), Polymerization initiator, Others	Shofu Dental Corporation	032370
SHOFU BLOCK HC	CAD/CAM hybrid ceramic restorative material	Silica powder, Zirconium silicate, UDMA, TEGDMA, Micro fumed silica, Pigments and others	Shofu Dental Corporation	0919982

*Bis-GMA* Bisphenol-A-diglycidyl methacrylate, *TEGDMA* Triethyleneglycol dimethacrylate, *Bis-MPEPP* Bisphenol A polyethoxy methacrylate, SiO_2_ silicon dioxide, Al_2_O_3_ aluminum oxide, *UDMA* urethane dimethacrylate, *NPGDMA* Neopentyl Glycol Dimethacrylate, *2-HEMA* 2-hydroxylethyl methacrylate

### Field isolation and cavity Preparation

Following local anesthesia administration, the operating field was isolated using rubber dam sheets (Sanctuary Dental Dam, Sanctuary Health SDN BHD), clamps (KSK Clamps, Dentech Corporation), and dental floss ligatures. Cavity preparation was performed using pear-shaped diamond burs (830 and 835, Komet USA), and carious dentin was removed with sharp excavators (Excavator Double Ended, Dentsply Maillefer). The cavity walls were shaped to achieve a 6–12° occlusal divergence using a round-end tapered diamond bur (850, Komet USA) [15]. A caliper was used to measure the base of each cusp to determine the need for coverage. Weakened cusps were reduced using a round-edge diamond wheel (909, Komet USA) [16].

Prepared cavities met the following criteria: remaining wall thickness of ≥ 1.5 mm; pulpal floor depth maintained at 1.5–2 mm for adequate restoration thickness; isthmus width of 2–3 mm; inter-occlusal clearance with functional cusps reduced by 2 mm and non-functional cusps by 1.5 mm; rounded internal line and point angles; and butt-joint cavo-surface margins [17]. Finally, the cavities were refined using yellow-coded diamond burs (858EF, Komet USA) [15, 16].

### Immediate dentin sealing and cavity optimization

For immediate dentin sealing, the prepared cavity was gently air-dried before applying a universal adhesive (BeautiBond Xtreme, Shofu Dental Corporation) to all dentin surfaces. The adhesive was actively rubbed for 20 s, gently air-thinned for 3 s, followed by a strong air-blow to obtain a glossy surface with no visible adhesive movement. Light curing was performed for 10 s using an LED light-curing device (Elipar S10, 3 M ESPE) at an intensity of 1200 mW/cm². Subsequently, the cavity was optimized with an injectable resin composite (Beautifil Flow Plus X F03, Shofu Dental Corporation) to block internal undercuts and prevent excessively thick restorations that might impair light transmission during cementation [17]. In cases of deep cervical margins without biological width violation, the margins were coronally elevated. The material was applied up to the dentin-enamel junction, and light cured for 20 s. Post-curing with glycerin gel was performed for an additional 20 s. Afterwards, enamel margins were reprepared with a finishing diamond bur [18].

### Final impression, bite registration and scanning

Final impressions were obtained using a two-step putty-wash technique with vinyl polysiloxane (A-silicone) impression materials (Panasil Putty and Panasil Initial Contact, Kettenbach GmbH & Co.) [19]. An alginate impression (Hydrogum, Zhermack SpA) for the opposing arch was taken, and bite registration was recorded (Charm Flex^®^ Bite Clear, DentKist Inc.). Provisional restorations were fabricated using a light-cured, eugenol-free temporary filling material (Applic, Maquira) and placed into the prepared cavities [20]. Master casts were scanned (InEos X5, Dentsply Sirona), obtaining multi-level HD scans of the upper and lower arches and the bite registration. The scanned data were converted into CAD files for digital design [19, 20].

### Computer-aided designing and milling (CAD/CAM)

Onlay restorations were designed using ExoCAD software (DentalCAD 3.1 Rijeka, Exocad GmbH). The internal cement gap was set at 80 μm, and the marginal gap at 0 μm [21]. Occlusal anatomy was adjusted, and both proximal and occlusal contacts were set to 0 μm [22]. Finally, the completed design was exported for milling. The onlays were fabricated using a milling machine (inLab MC X5, CEREC 3D, Dentsply Sirona) from hybrid ceramic blocks (SHOFU Block HC, Shofu Dental Corporation) [20, 23].

### Surface treatment protocol

After try-in, the intaglio surface of the restorations was sandblasted with 53 μm aluminum oxide (AquaCare, Velopex International) at 3 bar for 10 s, then placed in an ultrasonic cleaner with distilled water for 5 min [17]. Afterwards, the intaglio surface was cleaned with alcohol and dried with water- and oil-free air [16, 23]. Tooth-surface treatment was performed under rubber dam isolation. After removal of temporary restorations, the cavity surfaces were air-abraded with 53 μm aluminum oxide. A 37% phosphoric acid gel (Ventura etching gel, Madespa, S.A) was applied to enamel margins for 15 s and to cavity interiors for an additional 10 s, followed by thorough rinsing and air-drying [15, 17]. For the intervention group, a universal adhesive was applied to both the intaglio surface and the tooth surface for 20 s, air-dried for 5 s, and left uncured before cementation [24–26].

### Cementation protocol

For the intervention group, an injectable resin composite (BEAUTIFIL Flow Plus X F03, Shofu Dental Corporation) was used for cementation, while the control group received a self-adhesive, dual-cure resin cement (BeautiCem SA, Shofu Dental Corporation). The material was applied to the intaglio surface of the restoration, which was then seated into the cavity and held in place under light pressure for 3–4 min. For the dual-cure resin cement, tack-curing was performed for 2 s to facilitate the removal of excess material using a sharp explorer occlusally and dental floss interproximally [14]. In contrast, for the injectable resin composite, excess material was removed using a microbrush prior to polymerization, without the need for tack curing [27]. Final polymerization was achieved by light-curing each surface for 40 s, followed by the application of glycerin gel and an additional 40 s of light-curing per surface [24–26]. Occlusal adjustments and finishing were completed using fine-grit, yellow-coded diamond burs (852EF, 368EF, Komet USA), followed by polishing of the restorations (Enhance Finishing System, Dentsply Sirona) [15].

### Outcome assessment

The modified USPHS criteria were used by two independent, blinded assessors to evaluate the restorations at baseline, and at 6, 12, and 18 months. The primary outcome was restoration integrity (Fracture and retention), and the secondary outcomes were marginal adaptation, marginal discoloration, surface texture, secondary caries, proximal contact, anatomic contour, and postoperative sensitivity. In the event of any disagreement, a discussion was held to reach a consensus. If no agreement could be reached, a third assessor was consulted to resolve the discrepancy. Restorations were classified as Alpha (A) for an ideal clinical condition, Bravo (B) for a clinically acceptable condition, or Charlie (C) for a clinically unacceptable condition, as seen in Table 2. Inter-examiner reliability was assessed using Cohen’s Kappa coefficient, which indicated excellent agreement (κ = 0.92).

**Table 2:** Modified United States Public Health Service (USPHS) criteria

Criteria	Score	Characteristic
Restoration integrity (Retention)	Alpha (A)	Restoration is present.
	Charlie (C)	Restoration is absent.
Restoration integrity (Fracture)	Alpha (A)	No fracture of restoration (no visible cracks/chipping)
	Bravo (B)	Hairline crack and/or minor chipping, not affecting the marginal integrity or proximal contact (intraorally repairable)
	Charlie (C)	Severe crack/chipping or bulk fracture of restoration (requires replacement)
Marginal adaptation	Alpha (A)	Restoration is closely adapted to the tooth. Explorer does not catch, and there is no visible crevice along the periphery of the restoration.
	Bravo (B)	Explorer catches and there is visible evidence of a crevice, which the explorer penetrates. Dentin and/or base is not exposed along the margin.
	Charlie (C)	Explorer penetrates a crevice defect that extends to the dentin enamel junction. Dentin and/or base is exposed along the margin.
Marginal discoloration	Alpha (A)	No visual evidence of marginal discoloration between the restoration and the adjacent tooth structure.
	Bravo (B)	Visual evidence of marginal discoloration at the tooth-restoration junction but has not penetrated along the restoration in a pulpal direction.
	Charlie (C)	Visual evidence of marginal discoloration at the tooth-restoration junction, and the discoloration has penetrated along the restoration in a pulpal direction.
Surface texture	Alpha (A)	Surface texture is smooth, similar to the surrounding enamel, as determined by means of a sharp explorer.
	Bravo (B)	Surface texture is slightly rough, similar to a white stone surface, or slightly rougher than the surrounding enamel.
	Charlie (C)	Surface texture is very rough, with pitting sufficiently coarse to inhibit the continuous movement of an explorer across the surface.
Secondary caries	Alpha (A)	No caries is present.
	Charlie (C)	Caries is present.
Proximal contact	Alpha (A)	Physiological proximal contact is present.
	Bravo (B)	Weak or light proximal contact (no indication of damage to tooth, gingiva or periodontium; >100 μm).
	Charlie (C)	Far too weak proximal contact with traumatization of gingiva and food impaction.
Anatomic form	Alpha (A)	Restoration is a continuation of the existing anatomic form.
	Bravo (B)	Surface concavity is evident, but the dentin or base is not exposed.
	Charlie (C)	Loss of restorative substance such that a surface concavity is evident, and the base and/or dentin is exposed.
Postoperative sensitivity	Alpha (A)	No postoperative sensitivity detected.
	Charlie (C)	Postoperative sensitivity detected.

### Statistical analysis

Statistical analysis was conducted using IBM SPSS statistics software, version 2.1 for Windows. Qualitative data were presented as frequencies and percentages, while quantitative data were expressed as means and standard deviations. Data normality was assessed using the Kolmogorov-Smirnov and Shapiro-Wilk tests. The significance level was set at *P* ≤ 0.05. For demographic data, intergroup comparisons were performed using the Chi-squared test for qualitative variables and the independent Student’s t-test for quantitative variables. For clinical performance, intergroup comparisons were conducted using Chi-Squared test while intragroup comparisons were conducted using Cochran’s Q test. Kaplan–Meier analysis and the log-rank test were used to evaluate survival rates, with downgrades in USPHS scores (from Alpha to Bravo or Charlie) defined as events. A 95% confidence interval and 80% statistical power were applied, and all tests were two-tailed.

## Results

**Fig. 1:**
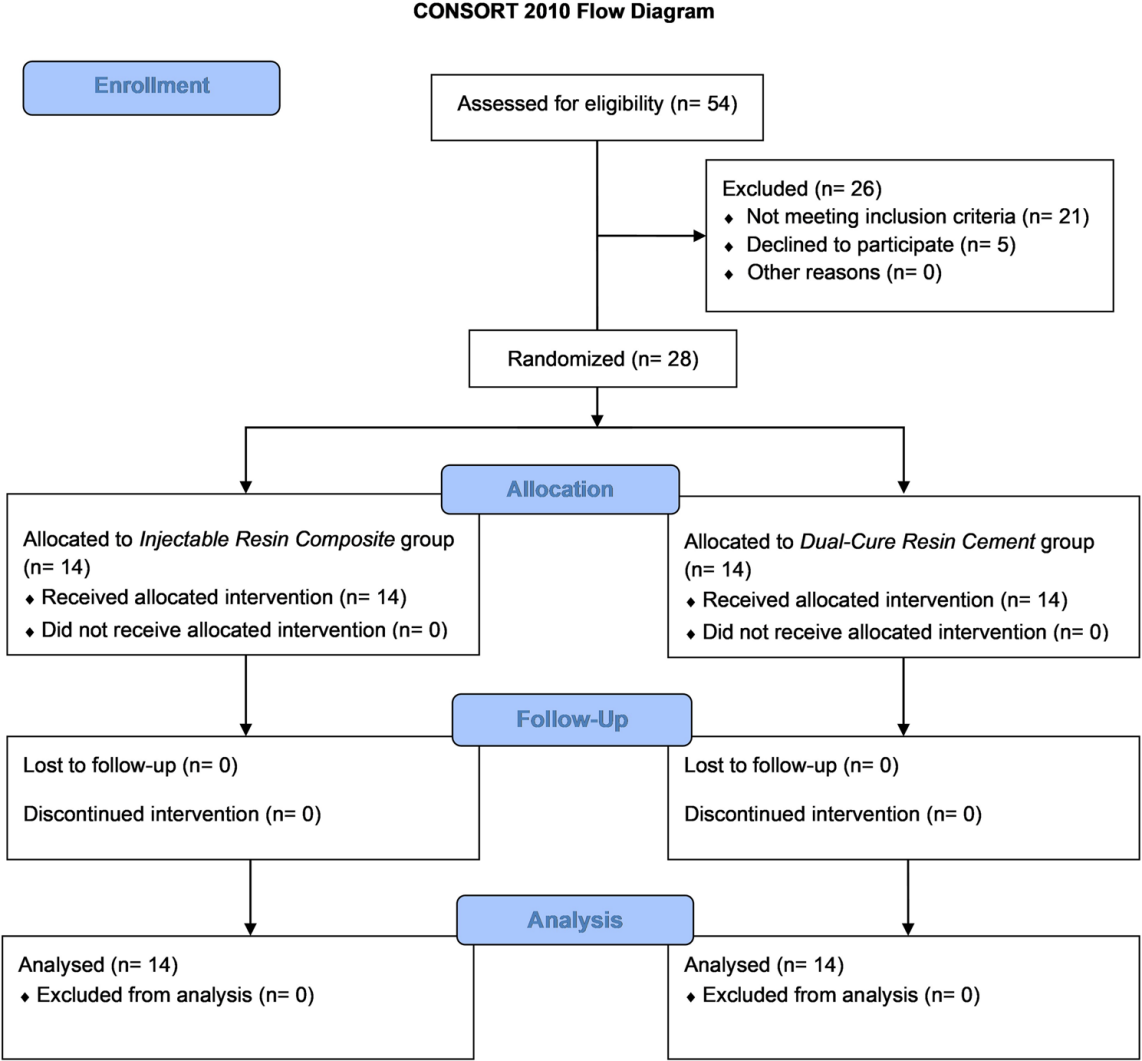
CONSORT 2010 Flow Diagram

All 28 participants completed the 18-month follow-up, yielding a 100% retention rate (Fig. 1). No statistically significant differences were observed between the two groups concerning demographic characteristics, including age (*P* = 0.314) and gender (*P* = 0.352), as shown in Table 3.

**Table 3:** Demographic characteristics of the study participants in both groups.

Demographic data		Total (*n* = 28)	Injectable resin composite (*n* = 14)	Dual-cure resin cement (*n* = 14)	*P*-value
Age (years)		29.53 ± 4.60	30.42 ± 4.79	30.43 ± 4.80	0.314NS
Gender	Male	12 (42.86%)	7 (50%)	5 (35.72%)	0.352NS
	Female	16 (57.14%)	7 (50%)	9 (64.28%)	

NS: non-significant at *P* > 0.05

Regarding clinical performance, intergroup comparisons revealed no significant differences for most outcomes (*P* > 0.05) across different follow-up periods. However, for marginal discoloration, injectable resin composite demonstrated superior performance. After 18 months, dual-cure resin cement had a higher incidence of marginal discoloration compared to injectable resin composite (*P* = 0.038). Intragroup comparisons revealed no significant changes for most outcomes (*P* > 0.05), except for marginal discoloration, which showed significant changes over time in the dual-cure resin cement group (*P* = 0.007).

**Fig. 2:**
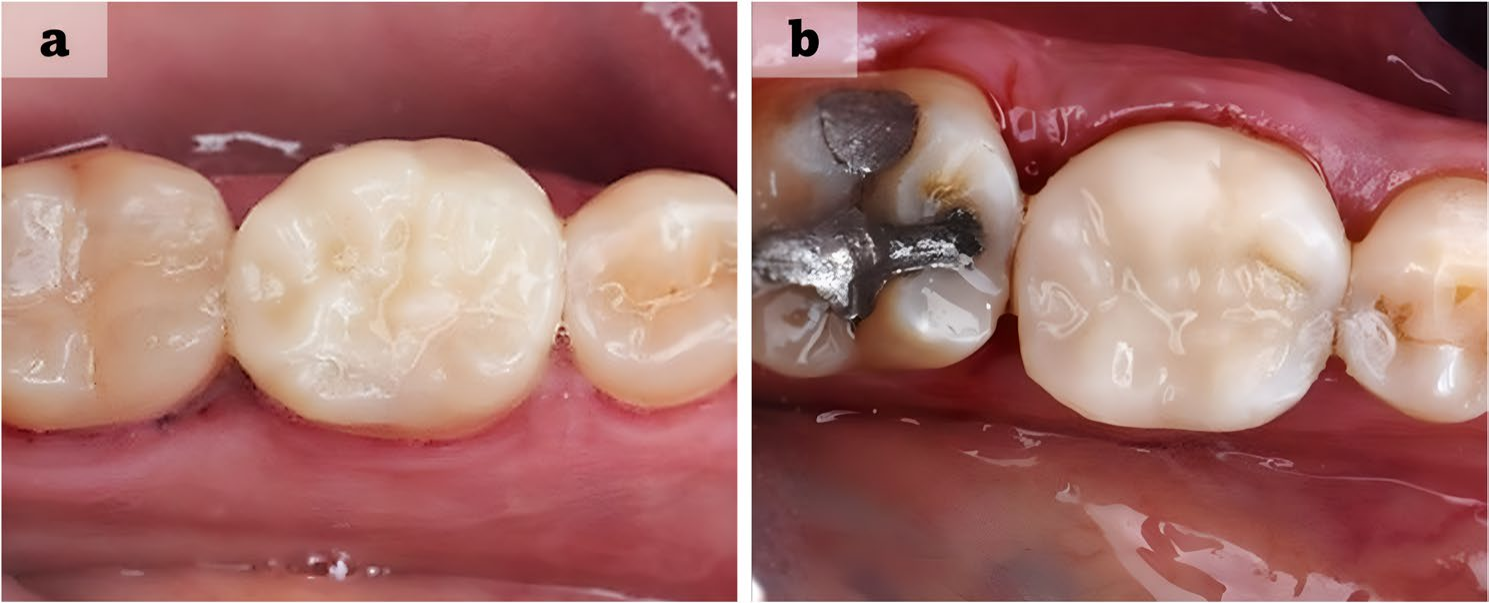
Postoperative photographs of indirect hybrid ceramic onlay restorations in mandibular first molars at the 18-month follow-up. (a) Injectable Resin Composite Group; (b) Dual-Cure Resin Cement Group, showing marginal discoloration

**Fig. 3:**
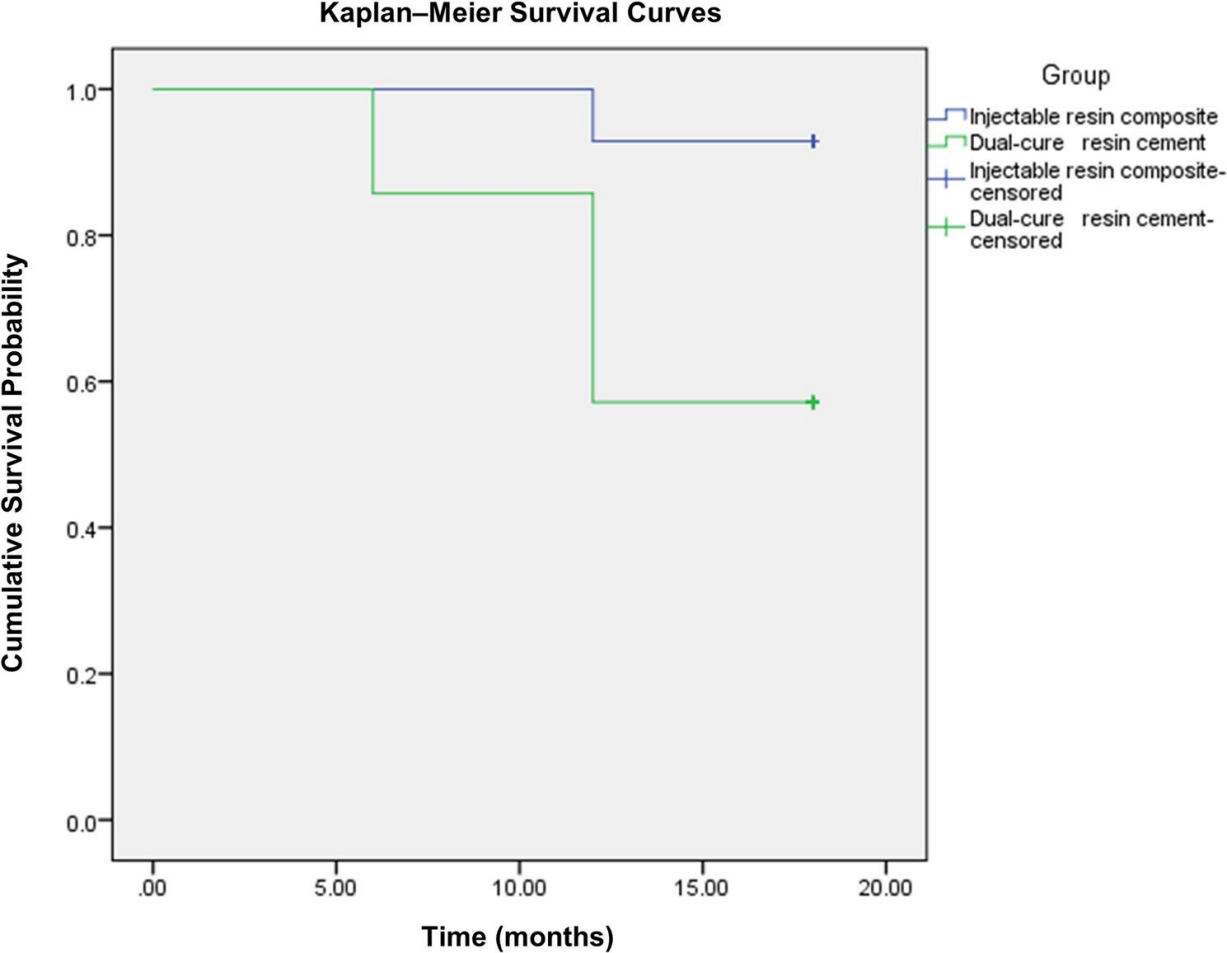
Kaplan–Meier survival curves comparing the injectable resin composite and dual-cure resin cement groups over an 18-month period

Regarding survival analysis, no restorations failed (Charlie score) in either group over the 18-month follow-up period. At 18 months, 92.86% of restorations in the injectable resin composite group received an Alpha score (clinically excellent) for marginal discoloration, compared to 57.14% in the dual-cure resin cement group (Fig. 2). Nevertheless, all restorations were classified as clinically successful (Table 4). Kaplan–Meier survival curves and the log-rank test demonstrated a statistically significant difference (*P* = 0.029) between the two groups (Fig. 3).

**Table 4.** Frequency and percentage of USPHS criteria scores showing intergroup and intragroup comparisons for all tested outcomes at different follow-up periods

Outcome	Follow-up	Injectable resin composite		Dual-cure resin cement		P-value
		Alpha	Bravo	Alpha	Bravo	
Restoration integrity (Fracture and Retention)	Baseline	14 (100%)	0 (0%)	14 (100%)	0 (0%)	1.000
	6 months	14 (100%)	0 (0%)	14 (100%)	0 (0%)	1.000
	12 months	14 (100%)	0 (0%)	14 (100%)	0 (0%)	1.000
	18 months	14 (100%)	0 (0%)	14 (100%)	0 (0%)	1.000
	P-value	1.000		1.000		
Marginal adaptation	Baseline	14 (100%)	0 (0%)	14 (100%)	0 (0%)	1.000
	6 months	14 (100%)	0 (0%)	14 (100%)	0 (0%)	1.000
	12 months	14 (100%)	0 (0%)	14 (100%)	0 (0%)	1.000
	18 months	13 (92.85%)	1 (7.15%)	12 (85.71%)	2 (14.29%)	0.5
	P-value	0.392		0.112		
Marginal discoloration	Baseline	14 (100%)	0 (0%)	14 (100%)	0 (0%)	1.000
	6 months	14 (100%)	0 (0%)	12 (85.71%)	2 (14.29%)	0.241
	12 months	13 (92.85%)	1 (7.15%)	10 (71.42%)	4 (28.58%)	0.163
	18 months	13 (92.85%)	1 (7.15%)	8 (57.14%)	6 (42.86%)	0.038*
	P-value	0.392		0.007*		
Surface texture	Baseline	14 (100%)	0 (0%)	14 (100%)	0 (0%)	1.000
	6 months	14 (100%)	0 (0%)	14 (100%)	0 (0%)	1.000
	12 months	14 (100%)	0 (0%)	14 (100%)	0 (0%)	1.000
	18 months	14 (100%)	0 (0%)	14 (100%)	0 (0%)	1.000
	P-value	1.000		1.000		
Secondary caries	Baseline	14 (100%)	0 (0%)	14 (100%)	0 (0%)	1.000
	6 months	14 (100%)	0 (0%)	14 (100%)	0 (0%)	1.000
	12 months	14 (100%)	0 (0%)	14 (100%)	0 (0%)	1.000
	18 months	14 (100%)	0 (0%)	14 (100%)	0 (0%)	1.000
	P-value	1.000		1.000		
Anatomic form	Baseline	14 (100%)	0 (0%)	14 (100%)	0 (0%)	1.000
	6 months	14 (100%)	0 (0%)	14 (100%)	0 (0%)	1.000
	12 months	14 (100%)	0 (0%)	14 (100%)	0 (0%)	1.000
	18 months	14 (100%)	0 (0%)	14 (100%)	0 (0%)	1.000
	P-value	1.000		1.000		
Proximal contact	Baseline	14 (100%)	0 (0%)	14 (100%)	0 (0%)	1.000
	6 months	14 (100%)	0 (0%)	14 (100%)	0 (0%)	1.000
	12 months	14 (100%)	0 (0%)	14 (100%)	0 (0%)	1.000
	18 months	14 (100%)	0 (0%)	14 (100%)	0 (0%)	1.000
	P-value	1.000		1.000		
Postoperative sensitivity	Baseline	14 (100%)	0 (0%)	14 (100%)	0 (0%)	1.000
	6 months	14 (100%)	0 (0%)	14 (100%)	0 (0%)	1.000
	12 months	14 (100%)	0 (0%)	14 (100%)	0 (0%)	1.000
	18 months	14 (100%)	0 (0%)	14 (100%)	0 (0%)	1.000
	P-value	1.000		1.000		

*: significant at *P* ≤ 0.05

## Discussion

This trial assessed the 18-month clinical performance of hybrid ceramic onlays luted with injectable resin composite versus dual-cure resin cement. Both materials demonstrated clinically acceptable and comparable outcomes for most parameters. However, marginal discoloration was significantly higher in the dual-resin cement group. Therefore, the null hypothesis is partially rejected, as a difference was observed in one clinical parameter.

The cementation procedure substantially influences the overall performance of indirect restorations. This intermediate, flexible layer plays a pivotal role in enhancing load-bearing capacity, optimizing stress distribution, and increasing fracture resistance, thereby reinforcing the underlying tooth structure and contributing to the restoration’s long-term clinical success [27]. Dual-cure resin cements have long been considered the gold standard for the cementation of indirect restorations. However, their inherent limitations—such as lower filler content and restricted working time—highlight the need for exploring alternative materials. Injectable resin composites, particularly those formulated with advanced filler technology and optimized viscosity, have emerged as potential candidates for indirect luting applications. Their use aligns with the growing interest in simplifying cementation protocols while enhancing both esthetic and mechanical outcomes.

Both groups exhibited favourable outcomes in the cementation of indirect restorations. Injectable composites demonstrate reliable adhesion when used with an appropriate adhesive system, ensuring durable bonding at the tooth-restoration interface [13]. These findings align with previous studies [12, 27, 29–32], which advocate the use of low-viscosity restorative resin composites for luting indirect restorations. These materials offer several clinical advantages, including an optimal balance between mechanical strength and minimal film thickness [30]. They help prevent air entrapment, which may occur with dual-component resin cements, and facilitate the removal of excess material prior to polymerization [8, 27]. Moreover, their handling properties make them suitable for both experienced and less-experienced clinicians, providing sufficient working time, particularly around preparation margins, thus enhancing procedural accuracy [31].

Similarly, dual-cure resin cements achieve a higher degree of conversion when both activation modes are effectively utilized, resulting in superior physicochemical and mechanical properties [7]. These findings are supported by a recent systematic review [33], which concluded that self-adhesive resin cements are effective for luting indirect single-unit restorations.

Marginal integrity is crucial for the long-term success of indirect restorations, as increased marginal gaps can lead to discoloration, microleakage, and secondary caries [2]. In the present study, both groups demonstrated satisfactory marginal adaptation. This comparable performance may be attributed to the adhesive properties of both materials, which help establish a reliable seal at the tooth-restoration interface [4, 29]. Moreover, their high flow characteristics promote effective penetration into the surface irregularities of the pretreated restoration, enhancing adaptation and minimizing the risk of incomplete seating and marginal discrepancies [1, 34]. However, a significant difference in marginal discoloration was observed between the two groups after 18 months, with dual-cure resin cement exhibiting a higher incidence. Marginal discoloration may arise from factors such as incomplete polymerization, material composition, water sorption, and resin matrix degradation [35].

Concerns have been raised regarding the use of light-cured resin composites as cements, particularly when light penetration is compromised, which may affect marginal discoloration and adaptation. However, systematic reviews have reported that light-cured cements exhibited a superior degree of conversion compared to dual-cured cements in restorations up to 2 mm thickness [3, 36]. This is consistent with the parameters applied in this study, where minimally invasive preparations were adopted through cavity optimization to ensure that the final restoration thickness did not exceed 2 mm. The limited thickness allowed sufficient light transmission and adequate polymerization in both groups. Furthermore, it has been emphasized that discrepancies in the degree of conversion between light-cure and dual-cure cements are more likely attributed to variations in cement composition rather than activation mode [3, 36].

Several studies have suggested that factors such as composition, type of photoinitiators, filler volume, and dispensing mode influence the degree of conversion of cements [37–39]. Supporting these findings, some studies have reported that self-adhesive cements exhibit a lower degree of conversion compared to conventional resin cements. This difference may be attributed to the higher viscosity of self-adhesive cements, which can impede radical mobility and reduce pH, potentially interfering with polymerization kinetics [39–41].

Another explanation for the discoloration tendency of dual-cure resin cements is their relatively low filler content, which may increase volumetric polymerization shrinkage [5, 42]. This could lead to a coefficient of thermal expansion higher than that of tooth tissues, potentially compromising the interface. Consequently, the cement may become exposed to the oral environment, adversely affecting both the long-term integrity and esthetics of the restoration [27].

Another potential drawback of dual-cure resin cements is their higher hydrophilicity compared to restorative resin composites, which may lead to increased water sorption and subsequent discoloration [42, 43]. Studies have shown that self-adhesive resin cements are more susceptible to water sorption, particularly due to their monomer content [43, 45]. This may help explain the findings of our study, where, in contrast to the hydrophilic nature of dual-cure resin cement, injectable resin composite not only exhibit greater hydrophobicity but was also used in combination with a HEMA-free adhesive. HEMA-free adhesive formulations, particularly those containing carboxylic and phosphoric acid monomers, have been shown to improve bonding durability and ensure more stable adhesive performance [29].

Our results are aligned with several studies that reported enhanced color stability of light-cure flowable composites compared to dual-cure resin cements [7, 46–48]. The discoloration observed in dual-cure cements has been attributed to the oxidation of initiators, the presence of unreacted tertiary amines, and the incorporation of benzoyl peroxide during photoactivation, all of which contribute to yellowing of the material and compromising esthetics [36, 46, 47]. Furthermore, a recent systematic review concluded that dual-polymerizing cements exhibit greater color variation than light-polymerized cements [49]. They recommended the use of light-polymerizable resin cements for luting thin ceramic restorations to minimize the risk of color change, as these cements contain lower concentrations of tertiary amines, a key factor contributing to their superior color stability compared to dual-cure resin cements.

Therefore, complementing the aforementioned findings, the superior performance of injectable resin composites in terms of marginal discoloration can be attributed to their higher filler content, enhanced mechanical properties, and improved wear resistance, all of which help minimize marginal staining. Additionally, their thixotropic nature promotes intimate adaptation to cavity walls, reducing the risk of microleakage, a primary contributor to marginal staining [49].

The findings of this study revealed that no secondary caries or postoperative sensitivity were observed in either group during the 18-month follow-up period. Several factors likely contributed to these favourable outcomes, including the use of rubber dam isolation, precise restoration fabrication through CAD/CAM technology, and the implementation of immediate dentin sealing. Recent systematic reviews recommend sealing dentin surfaces immediately after preparation to prevent or reduce postoperative sensitivity [50–52]. Both materials used in this study are bioactive, incorporating proprietary S-PRG fillers that actively release six beneficial ions. These ions inhibit bacterial adhesion, stabilize oral pH, reduce the risk of secondary caries, and promote remineralization [53]. These properties may have contributed to the favourable outcomes observed in this trial and may offer continued protection against recurrent caries in the long term.

To our knowledge, this is the first clinical trial to evaluate injectable resin composites as viable alternatives to dual-cure resin cements for the luting of indirect restorations. However, certain limitations should be acknowledged, including the relatively small sample size, the 18-month follow-up period, and the single-center design. Future studies with larger cohorts and extended follow-up durations are necessary to further clarify the long-term behaviour of injectable resin composites in this context. Additionally, multicenter trials involving a broader range of practitioners and patient populations are recommended to improve the generalizability and clinical relevance of the findings. Further research should also assess the applicability of injectable resin composites across diverse clinical scenarios, including comparisons with light-cured resin cements and their use with different types of indirect restorations.

## Conclusions

Under the limitations of this trial, it can be concluded that:

1.  Injectable resin composites represent a clinically promising alternative to dual-cure resin cements for luting indirect onlay restorations. Their comparable performance across most clinical parameters supports their potential integration into routine adhesive workflows, particularly in cases where simplified handling and enhanced mechanical properties are desired.
2.  The superior marginal esthetic performance observed with injectable resin composites suggests a potential advantage in long-term color stability, making them especially suitable for esthetically demanding cases.
3.  Both cementation protocols demonstrated consistent and stable clinical outcomes, with no failures reported during the 18-month follow up, reinforcing the reliability of injectable resin composites as an effective cementation approach when properly executed.

## Data Availability

The data supporting the findings of this study are available from the corresponding author upon reasonable request.
